# Surgical Outcomes of Vaginal or Cervical Melanoma

**DOI:** 10.3389/fsurg.2021.771160

**Published:** 2022-01-03

**Authors:** Hui Tian, Xuan Wang, Bin Lian, Lu Si, Min Gao, Hong Zheng, Zhihong Chi, Yan Kong, Lili Mao, Xue Bai, Bixia Tang, Xieqiao Yan, Siming Li, Li Zhou, Jie Dai, Yangchun Sun, Lingying Wu, Jun Guo, Chuanliang Cui

**Affiliations:** ^1^Key Laboratory of Carcinogenesis and Translational Research (Ministry of Education), Department of Renal Cancer and Melanoma, Peking University Cancer Hospital and Research Institute, Beijing, China; ^2^Key Laboratory of Carcinogenesis and Translational Research (Ministry of Education), Department of Gynecologic Oncology, Peking University Cancer Hospital and Research Institute, Beijing, China; ^3^Department of Gynecologic Oncology, National Cancer Center/National Clinical Research Center for Cancer/Cancer Hospital, Chinese Academy of Medical Sciences and Peking Union Medical College, Beijing, China

**Keywords:** mucosal melanoma, gynaecological melanoma, surgical approach, radical resection, non-radical resection

## Abstract

**Objective:** To evaluate the effectiveness of radical resection compared with non-radical resection for vaginal or cervical melanoma.

**Methods:** We retrospectively analysed the clinical data of post-operative patients with primary lower genital tract melanoma hospitalised at Peking University Cancer Hospital between Jan 2014 and Dec 2020. The study endpoints were recurrence-free survival (RFS) and overall survival (OS). Kaplan–Meier method-plotted survival curves and univariate and multivariate Cox proportional hazards regression models were used to identify the factors associated with RFS and OS, and to calculate hazard ratios (HRs) and associated 95% confidence intervals (95% CIs).

**Results:** A total of 80 patients were included. Thirty-one patients had received non-radical resection, and 49 patients had received radical resection. The median patient age was 55.5 (IQR 45.3–60.0) years. Sixty-two (77.5%) patients had vaginal melanoma. Sixty-four patients (80.0%) had received post-operative adjuvant therapy. The median follow-up time was 36.0 months (95% CI 10.1–62.1 months). Sixty-four patients developed recurrence, and 44 patients died. The median RFS (mRFS) was 6.0 months (95% CI 3.4–8.6 m), and the RFS for the radical resection group was longer than that for the non-radical resection group (9.5 vs. 5.3 m), with no significant difference (*P* > 0.05). The median OS (mOS) was 25.9 months (95% CI 14.4–37.4 m). The mOS was 24.6 months (95% CI 10.3–38.9 m) and 25.9 months (95% CI 10.9–40.9 m) in the non-radical resection group and the radical resection group, respectively. Multivariate Cox regression analysis showed that surgical approach, infiltration depth of the tumour, lymph node metastasis, and post-operative adjuvant therapy were independent risk factors for RFS and that post-operative adjuvant therapy was an independent risk factor for OS.

**Conclusion:** By performing multivariate analysis, which corrected for potential confounding factors, we identified surgical procedures that were associated with RFS, and we found that RFS and OS in patients with vaginal melanoma and cervical melanoma benefitted from post-operative adjuvant therapy.

## Introduction

Mucosal melanoma is a rare melanoma subtype in the West, but it is the second most common subtype in Asia (1). Owing to its distinctive biological features, mucosal melanoma has a different clinical presentation and treatment from cutaneous melanoma. Surgical resection is the main treatment for early-stage melanoma because of its survival benefit (2). To date, the National Comprehensive Cancer Network (NCCN) has not established clinical practise guidelines for mucosal melanoma. Primary gynaecological melanoma is a rare aggressive malignant disease and the third most common subtype of mucosal melanoma, comprising 22.6% of all mucosal melanomas (3). Many surgical procedures for gynaecological melanoma exist, such as local tumour resection, wide tumour resection, and radical resection. Because gynaecological melanoma is an uncommon disease, randomised prospective clinical studies to assess the effectiveness of different surgical procedures are lacking. The available evidence regarding surgical treatment for gynaecological melanoma is from a series of retrospective studies of primary gynaecological melanoma and extrapolation from cutaneous melanoma (4–7). The purpose of this study was to evaluate the effectiveness of radical resection compared with non-radical resection for gynaecological melanoma.

## Materials and Methods

### Study Design and Patients

We retrospectively analysed clinical data of post-operative patients with primary lower genital tract melanoma hospitalised at Peking University Cancer Hospital between Jan 2014 and Dec 2020. Patients were included based on the following criteria: (1) pathological confirmation of primary lower genital tract mucosal melanoma at Peking University Cancer Hospital; (2) treatment with non-radical resection or radical resection to fully remove the tumour and no neoadjuvant chemotherapy; and (3) a follow-up time of more than half a year. Non-radical resection included local tumour excision, wide tumour excision, and partial vaginectomy with or without complete lymph node dissection (CLND); radical resection included radical vaginectomy, radical hysterectomy with or without radical parametrectomy, and with or without CLND. CLND included pelvic node dissection, aortic node dissection, and unilateral or bilateral inguinal lymph node dissection. The exclusion criteria were as follows: (1) treatment with debulking dissection that had only partially removed the tumour; (2) type of surgery unknown; (3) surgery recorded unknown; (4) coexistence of any other primary tumour; and (5) distant metastasis at diagnosis.

A total of 80 patients were included in this study. The patients were divided into the non-radical resection group and the radical resection group based on the operation performed. The following clinical data were collected: patient demographic data (including age, genetic mutation status, lymph node status, etc.), histopathology of the tumour (including the primary site, infiltration depth, ulceration, etc.), treatment, and outcomes. Because the cancer staging criteria for cutaneous melanoma in the American Joint Committee on Cancer (AJCC) staging manual are not recommended for mucosal melanoma (8), we collected the infiltration depth of the tumour, the status of the ulceration, and the status of lymph nodes to describe tumour burden.

The study endpoints were recurrence-free survival (RFS) and overall survival (OS). RFS was defined as the time (in months) from the date of operation to disease recurrence, and OS was defined as the time (in months) from the date of operation to death from any cause.

### Statistical Analysis

Differences in continuous variables, such as age, were examined by *t*-tests. Differences in categorical variables were examined by the chi-square test or Fisher's exact test, if appropriate. RFS and OS curves were plotted using the Kaplan–Meier method and compared between groups using the log-rank test. Furthermore, univariate and multivariate Cox proportional hazards regression models were used to identify the factors associated with RFS and OS and to calculate hazard ratios (HRs) and associated 95% confidence intervals (95% CIs). Subgroup analyses according to baseline clinical characteristics were performed to compare the effectiveness of non-radical resection and radical resection with respect to RFS in different subgroups through the univariate Cox regression model. All analyses were performed with SPSS, version 23 (IBM Corp., Armonk, NY), and GraphPad PRISM, version 6 (GraphPad Software, LLC). All tests were two-sided, with *P*-values < 0.05 considered statistically significant.

## Results

### Baseline Clinical Characteristics

A total of 80 patients met the inclusion criteria. Table 1 lists patient demographics and tumour characteristics. The median patient age was 55.5 (IQR 45.3–60.0) years. Sixty-two (77.5%) patients had vaginal melanoma. The majority of patients (93.8%) did not have lymph node metastasis. Three commonly mutated genes, BRAF, RAS and KIT, tested wild type in nearly two-thirds of the patients. The most common infiltration depth of the tumour was the mucosal layer (41.3%), and the infiltration depth of the tumour was unknown in thirty patients. Thirty-one patients had received non-radical resection, and forty-nine patients had received radical resection. The primary site, CLND, and infiltration depth of the tumour were significantly different between the two groups, and the other variables were balanced. Among the thirty-one patients in the non-radical resection group, patients with vaginal melanoma accounted for a large proportion (93.5%), and most patients (80.6%) were not treated with CLND; in nineteen (63.3%) of the patients in this group, the infiltration depth of the tumour was the mucosal layer. Among forty-nine patients in the radical resection group, two-thirds (67.3%) had vaginal melanoma, twelve (25.4%) had cervical melanoma, and four had melanoma of the vagina and cervix; more than half of the patients (53.1%) had received CLND; fourteen patients (28.6%) had involvement of the mucosal layer, and twenty-two patients (44.9%) had an unknown infiltration depth. Most patients (80.0%) had received post-operative adjuvant therapy.

**Table 1:** Patient demographics and tumour characteristics.

	**Specification**	**Total (*N* = 80)**	**Non-radical (*N* = 31)**	**Radical (*N* = 49)**	***P*-value**
Age, years	Median (IQR)	55.5 (45.3–60.0)	58 (49.0–62.0)	53.0 (45.0–59.0)	0.063
Primary site, *N* (%)	Vagina	62 (77.5)	29 (93.5)	33 (67.3)	0.014
	Cervix	14 (17.5)	2 (6.5)	12 (24.5)	
	Vagina+cervix	4 (5.0)	0	4 (8.2)	
CLND, *N* (%)	No	48 (60.0)	25 (80.6)	23 (46.9)	0.03
	Yes	32 (40.0)	6 (19.4)	26 (53.1)	
Mutation status, *N* (%)	BRAF/RAS/KIT wild type	51 (63.7)	22 (71.0)	29 (59.2)	0.285
	KIT	5 (6.2)	0	5 (10.2)	
	RAS	8 (10.0)	3 (9.7)	5 (10.2)	
	BRAF	7 (8.8)	3 (9.7)	4 (8.2)	
	Unknown	9 (11.3)	3 (9.7)	6 (12.2)	
Infiltration depth, *N* (%)	Mucosa	33 (41.3)	19 (63.3)	14 (28.6)	0.004
	Muscular layer	8 (10.0)	1 (3.2)	7 (14.3)	
	Serous membrane	5 (6.2)	2 (6.5)	3 (6.1)	
	Adjacent structures	4 (5.0)	1 (3.2)	3 (6.1)	
	Unknown	30 (37.5)	8 (25.8)	22 (44.9)	
Ulceration, *N* (%)	Yes	53 (66.2)	23 (74.2)	30 (61.2)	0.232
	No	15 (18.8)	6 (19.4)	9 (18.4)	
	Unknown	12 (15.0)	2 (6.5)	10 (20.4)	
LN metastasis, *N* (%)	No	75 (93.8)	29 (93.5)	46 (93.9)	0.249
	Yes	5 (6.2)	2 (6.5)	3 (6.1)	
Adjuvant therapy, *N* (%)	Yes	64 (80.0)	24 (77.4)	40 (81.6)	0.646
	No	16 (20.0)	7 (22.6)	9 (18.4)	

### Overall Analysis

**Figure 1:**
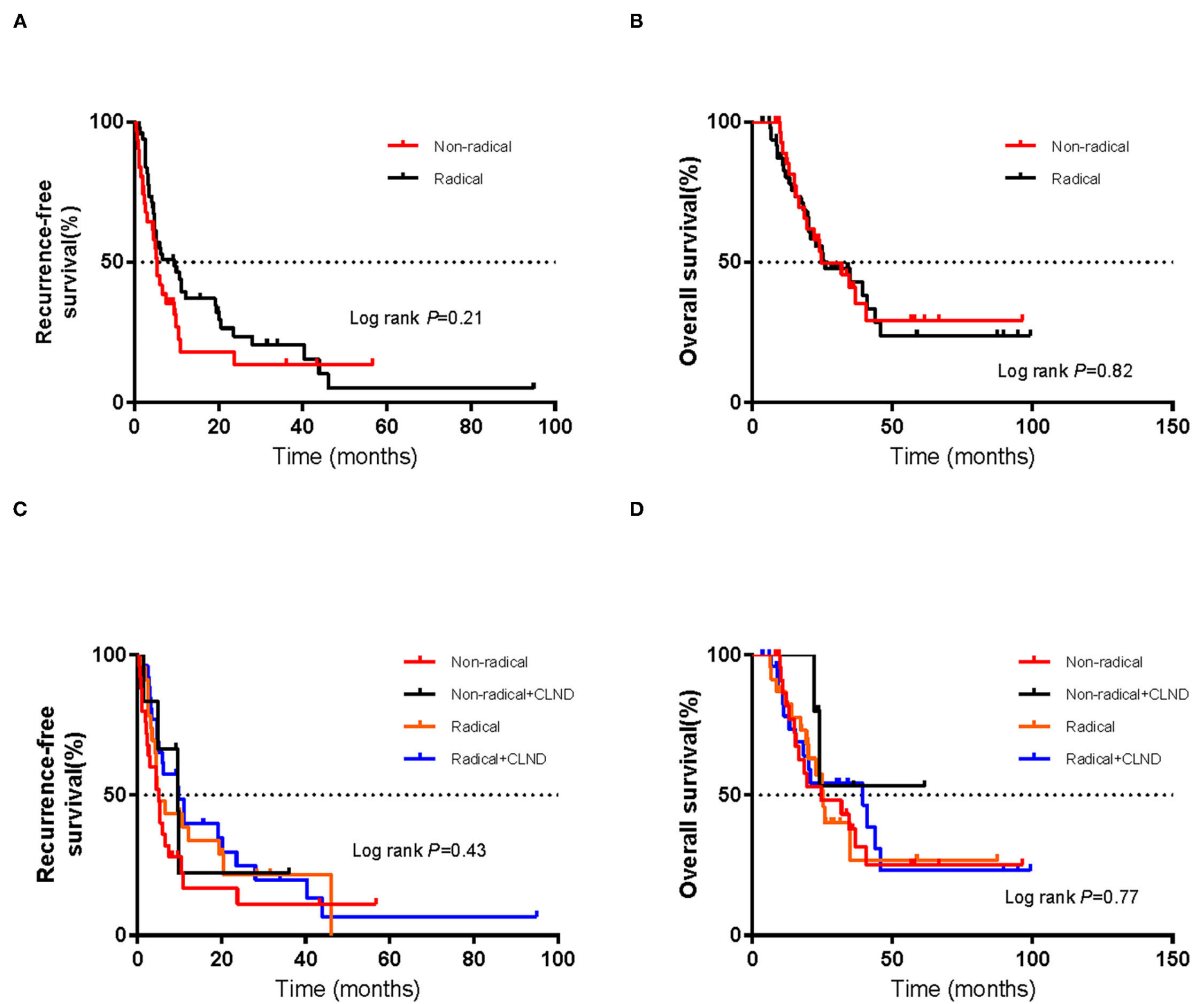
Kaplan–Meier curves of **(A)** recurrence-free survival and **(B)** overall survival in the non-radical resection and radical resection groups. Kaplan–Meier curves of **(C)** recurrence-free survival and **(D)** overall survival of patients who did or did not undergo complete lymph node dissection (CLND) in each of the non-radical resection and radical resection groups. The log-rank test was used to evaluate differences between groups.

As of July 1, 2021, the cut-off date of follow-up, the median follow-up time was 36.0 months (95% CI 10.1–62.1 m). As calculated by the Kaplan–Meier method, the median RFS and OS (mRFS and mOS) were 6.0 months (95% CI 3.4–8.6 m) and 25.9 months (95% CI 14.4–37.4 m), respectively. Sixty-four patients developed recurrence, and forty-four patients died. In the non-radical resection group, twenty-five patients developed first recurrence, and seventeen patients died. In contrast, in the radical resection group, thirty-nine patients, and twenty-seven patients died. Using the abovementioned method, the estimated mRFS was 5.3 months (95% CI 3.7–6.9) in the non-radical resection group and 9.5 months (95% CI 3.3–16.7) in the radical resection group. Although the RFS of the radical resection group was longer than that of the non-radical resection group, the difference was not significant (*P* > 0.05) (Figure 1A).

**Figure 2:**
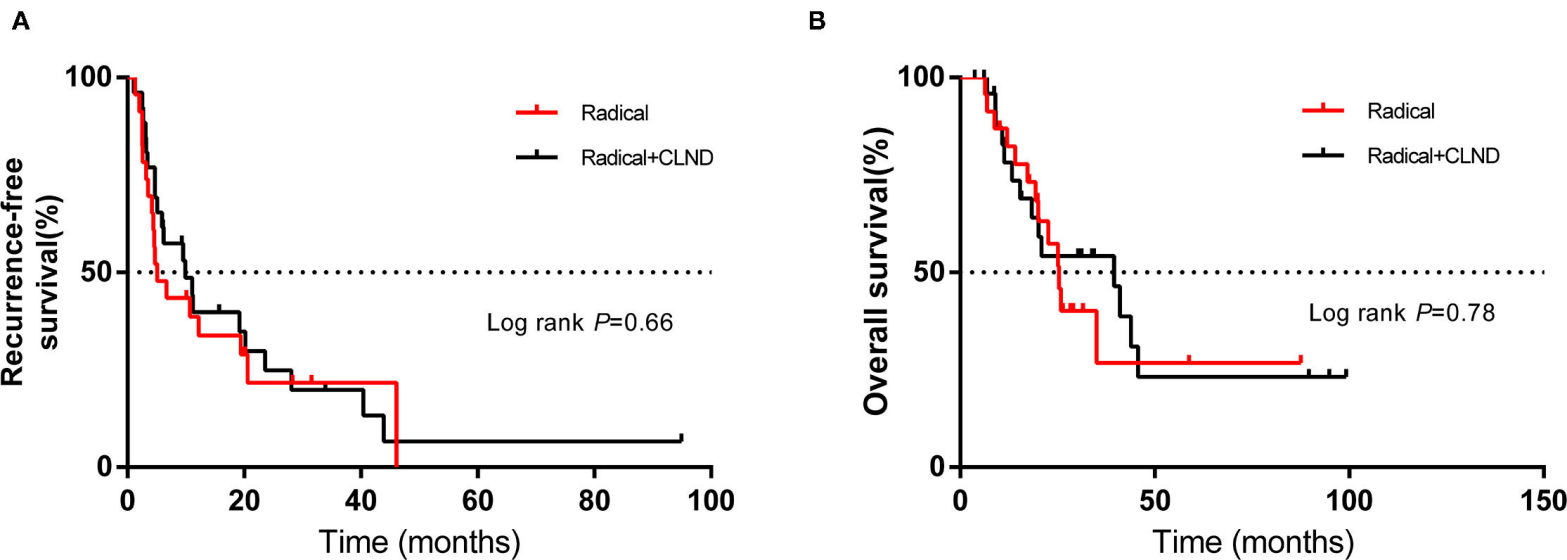
Kaplan–Meier curves of **(A)** recurrence-free survival and **(B)** overall survival of patients in the radical resection group who did or did not undergo complete lymph node dissection (CLND). The log-rank test was used to evaluate differences between groups.

Using the abovementioned method, the estimated mOS was 24.6 months (95% CI 10.3–38.9 m) for the non-radical resection group and 25.9 months (95% CI 10.9–40.9 m) for the radical resection group, with no significant difference (*P* > 0.05) (Figure 1B). RFS and OS for the two groups in which CLND was/was not performed were not significantly different (*P* > 0.05) (Figures 1C,D). Among the forty-nine patients in the radical resection group, more than half (53.1%) had undergone CLND. Within the radical resection group, the estimated mRFS was 9.9 months for the patients who underwent CLND (95% CI 2.6–17.2 months) and 5.1 months for those who did not (95% CI 1.8–8.4 m); the corresponding OS were 39.5 months (95% CI 16.0–63.0 m) and 25.4 months (95% CI 21.1–29.7 m), respectively. However, the differences were not significant (*P* > 0.05) (Figures 2A,B).

### Post-operative Complications

The post-operative complications were known for forty-two patients: nineteen patients in the non-radical resection group and twenty-three patients in the radical resection group. Five patients developed post-operative complications: two patients in the non-radical resection group and three patients in the radical resection group. Two patients in the non-radical resection group who underwent partial vaginectomy experienced delayed operative incision healing. Among the twenty-three patients in the radical resection group, one patient who received radical hysterectomy without CLND experienced delayed operative incision healing, one patient who received radical hysterectomy with CLND experienced bowel obstruction and urinary retention, and one patient who received radical vaginectomy experienced urinary retention.

### RFS and OS According to the Primary Site

**Figure 3:**
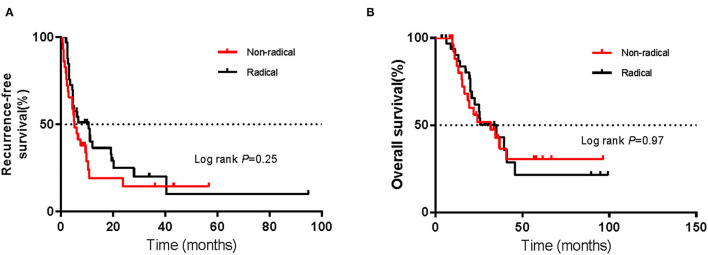
Kaplan–Meier curves of **(A)** recurrence-free survival and **(B)** overall survival of patients with vaginal melanoma in the non-radical resection and radical resection groups. The log-rank test was used to evaluate differences between groups.

Among the sixty-two patients with vaginal melanoma, thirty-three were in the radical resection group, while twenty-nine were in the non-radical resection group. As determined with the Kaplan–Meier method, mRFS was 5.3 months (95% CI 3.7–7.2 m) in the non-radical resection group and 10.7 months (95% CI 3.1–18.3 m) in the radical resection group. The mRFS of the radical resection group was twice as long as that of the non-radical resection group; nevertheless, the difference was not significant (*P* > 0.05) (Figure 3A). Additionally, mOS was 31.9 months (95% CI 13.4–50.4 m) and 35 months (95% CI 21.2–48.8 m) in the non-radical resection group and the radical resection group, respectively, with no significant difference (*P* > 0.05) (Figure 3B).

Of fourteen patients with cervical melanoma, most (12 patients) were in the radical resection group, with only 2 in the non-radical group. As estimated with the abovementioned method, mRFS was 0.4 months (95% CI NA) for the non-radical resection group and 5.9 months (95% CI 0–13.4 m) for the radical resection group, and mOS was 24.6 months (95% CI NA) and 18.4 months (95% CI 6.1–30.7 m) for the radical resection group and non-radical resection group, respectively.

### RFS and OS According to Medical Management

**Figure 4:**
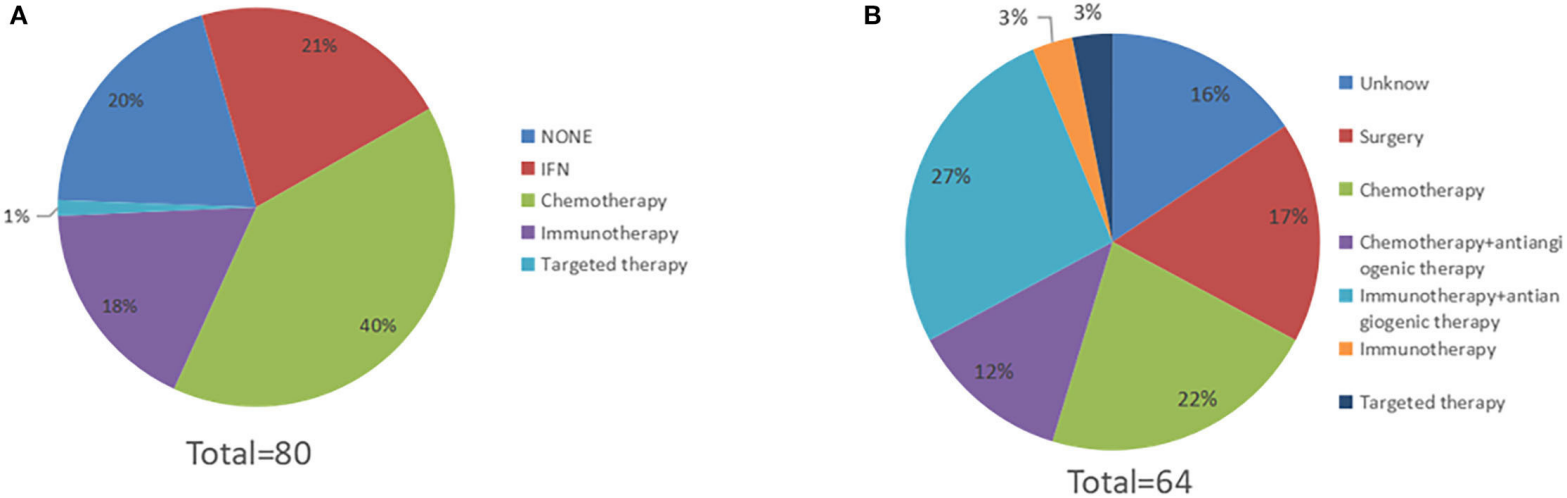
The spectrum of medical treatment. **(A)** Sixty-four patients received post-operative therapy, 17 patients received high-dose IFNa-2b, 30 patients received chemotherapy, 14 patients received immunotherapy, and 1 patient received targeted therapy. **(B)** Sixty-four patients experienced relapse. After relapse, 11 patients underwent reoperation, 14 patients received chemotherapy, 2 patients received immunotherapy, 2 patients received targeted therapy, 17 patients received immunotherapy combined with antiangiogenic therapy, and 8 patients received chemotherapy combined with antiangiogenic therapy (The treatments of 10 patients were unknown).

**Figure 5:**
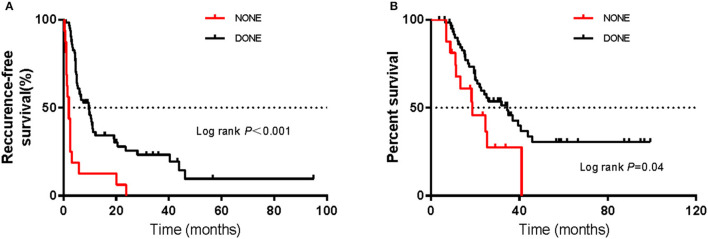
Kaplan–Meier curves of **(A)** recurrence-free survival and **(B)** overall survival in patients receiving or not receiving post-operative therapy. The log-rank test was used to evaluate differences between groups.

Among all patients, sixty-four had received post-operative adjuvant therapy, and sixteen had not received any post-operative treatment. Of sixty-four patients, seventeen had received high-dose IFNa-2b (HDI) treatment, thirty-two had received chemotherapy, fourteen had received immunotherapy, and one had received targeted therapy with c-KIT amplification (Figure 4A). Post-operative adjuvant therapy significantly prolonged RFS, with mRFS of 9.5 months (95% CI 5.7–13.3 m) and 2.0 months (95% CI 1.0–3.0 m) (*P* < 0.05) (Figure 5A), and significantly prolonged OS (*P* < 0.05) (Figure 5B).

A total of sixty-four patients experienced relapse. Among them, twenty-five patients experienced locoregional recurrence only: fifteen patients in the non-radical resection group and ten patients in the radical resection group. Thirty-two patients developed distant metastases only: nine patients in the non-radical resection and twenty-three patients in the radical resection. In addition, five patients experienced both distant metastasis and locoregional recurrence: one patient in the non-radical resection group and four patients in the radical resection group. The metastatic sites of two patients in the radical resection group who experienced relapse were unknown.

After relapse, eleven patients underwent reoperation. Of the eleven patients, nine patients experienced locoregional recurrence, one patient experienced metastasis of distant lymph nodes, and one patient developed oligometastasis in the breast. The mRFS of reoperation for the eleven patients was 4.4 months (95% CI 1.8–7.0 m). Fourteen patients received chemotherapy, two patients received immunotherapy, two patients received targeted therapy, eight patients received chemotherapy combined with antiangiogenic therapy, and seventeen patients received immunotherapy combined with antiangiogenic therapy (with the treatments of ten patients being unknown) (Figure 4B).

### Subgroup Analysis

We used univariate and multivariate Cox proportional hazards models to identify prognostic factors among the baseline characteristics. In the univariate Cox regression analysis, we estimated the association between the type of operation and RFS, and the HR for the radical resection group compared with the non-radical resection group was 0.72 (95% CI 0.44–1.21, *P* > 0.05). The results of the univariate analyses showed that several variables were associated with RFS, including infiltration depth of the tumour, lymph node metastasis, and adjuvant therapy (Table 2). Given the possibility of effects of possible confounders, we used a multivariate Cox proportional hazards model with the abovementioned factors to adjust the HR of the radical resection group; in the multivariate model, the HR of the radical resection group compared to the non-radical resection group was 0.55 (95% CI 0.31–0.98, *P* < 0.05). The type of operation, infiltration depth of the tumour, lymph node metastasis, and post-operative adjuvant therapy were identified as independent risk factors for RFS (Table 3).

**Table 2:** Results of univariate analyses of recurrence-free survival (RFS) and overall survival (OS).

**Characteristic**	**Specification**	**RFS**		**OS**	
		**HR (95% CI)**	***P*-value**	**HR**	***P*-value**
Age, years	Mean (IQR)	1.00 (0.98–1.03)	0.88	0.98 (0.95–1.01)	0.27
Primary site	Vagina	Reference		Reference	
	Cervix	1.10 (0.59–2.08)	0.76	1.79 (0.82–3.89)	0.14
	Vagina+cervix	0.88 (0.31–2.49)	0.81	0.72 (0.17–3.03)	0.66
Operation type	LTE	Reference		Reference	
	Radical	0.72 (0.44–1.20)	0.21	1.07 (0.58–1.97)	0.82
CLND	No	Reference		Reference	
	Yes	0.74 (0.45–1.23)	0.24	0.81 (0.44–1.50)	0.50
Mutation status	BRAF/RAS/KIT wild type	Reference		Reference	
	KIT	0.50 (0.15–1.63)	0.25	0.28 (0.04–2.06)	0.21
	RAS	1.54 (0.64–3.71)	0.34	1.44 (0.50–4.16)	0.50
	BRAF	0.85 (0.36–2.00)	0.70	0.67 (0.24–1.92)	0.46
	Unknown	1.12 (0.50–2.52)	0.78	1.75 (0.66–4.59)	0.26
Infiltration depth	Mucosa	Reference		Reference	
	Muscular layer	0.95 (0.39–2.33)	0.91	2.36 (0.83–6.73)	0.11
	Serous membrane	7.16 (2.46–20.87)	<0.001	2.39 (0.77–7.47)	0.13
	Adjacent structures	1.27 (0.44–3.67)	0.66	2.67 (0.86–8.33)	0.09
	Unknown	1.19 (0.68–2.09)	0.55	1.53 (0.74–3.16)	0.25
Ulceration	No	Reference		Reference	
	Yes	0.99 (0.52–1.87)	0.96	0.82 (0.40–1.70)	0.60
	Unknown	0.55 (0.22–1.34)	0.19	0.60 (0.22–1.66)	0.32
LN metastasis	No	Reference		Reference	
	Yes	2.93 (1.16–7.40)	0.02	1.73 (0.53–5.70)	0.37
Adjuvant therapy	No	Reference		Reference	
	Yes	0.25 (0.14–0.45)	<0.001	0.49 (0.24–0.98)	0.04

**Table 3:** Results of multivariate analyses of recurrence-free survival (RFS).

**Characteristic**	**Specification**	**HR (95% CI)**	***P*-value**
Operation type	Non-radical	Reference	0.04
	Radical	0.55 (0.31–0.98)	
Infiltration depth	Mucosa	Reference	0.004
	Muscular layer	1.45 (0.56–3.89)	
	Serous membrane	8.31 (2.74–25.19)	
	Adjacent structures	2.34 (0.77–7.15)	
	Unknown	1.94 (0.98–3.84)	
LN metastasis	No	Reference	0.008
	Yes	3.73 (1.41–9.85)	
Adjuvant therapy	No	Reference	<0.001
	Yes	0.21 (0.11–0.40)	

*P-value for the interaction*.

**Figure 6:**
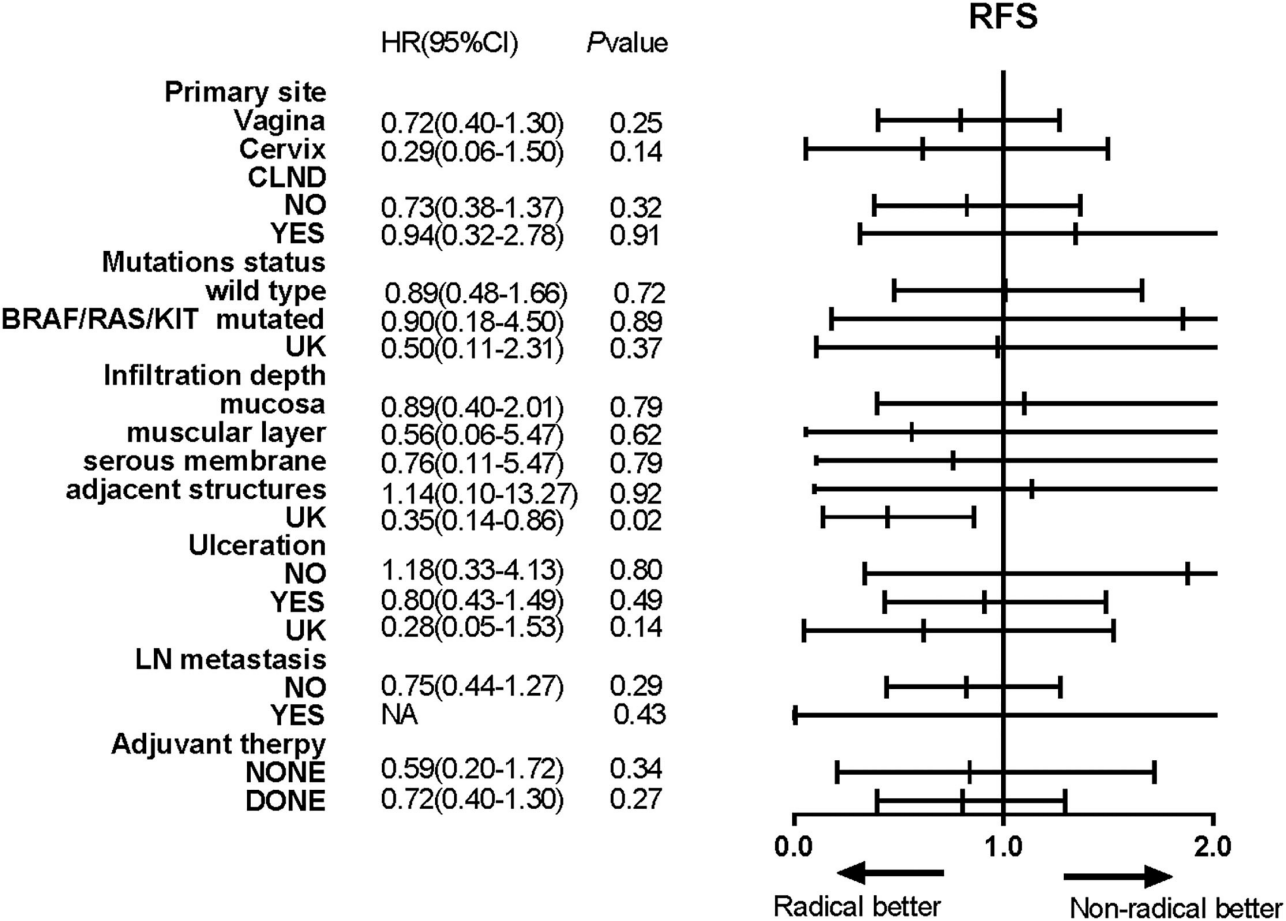
Forest plots showed the results of univariate analyses of recurrence-free survival (RFS) in different subgroups. Hazard ratios (HRs), their associated 95% confidence intervals (95% CIs) and *P*-values were calculated for each subgroup comparison.

Univariate Cox regression analyses conducted using the abovementioned method showed that the infiltration depth of the tumour and post-operative adjuvant therapy also were correlated with OS (Table 2), and the HR for the radical resection group compared with the non-radical resection group was 1.07 (95% CI 0.58–1.97, *P* > 0.05). The multivariate Cox proportional hazards models showed that post-operative adjuvant therapy was an independent risk factor for OS, and the type of operation was not associated with OS (*P* = 0.77). Univariate Cox regression analyses did not reveal any between-group differences in RFS in subgroups stratified by baseline characteristics (*P* > 0.05, Figure 6).

## Discussion

Mucosal melanoma originates mainly from melanocytes in mucosal membranes. Primary gynaecological melanoma is rare, comprising 22.6% of all mucosal melanomas (3). Gynaecological melanoma has an insidious onset and is associated with a poor prognosis (9). At the early stage, complete resection of the primary tumour is vital (2, 10). However, at present, no standard surgical procedure has been established.

In our study, by constructing multivariate Cox proportional hazards models, we found that surgical approach was an independent risk factor for RFS but not OS (Table 2). Given the rarity of gynaecological melanoma, few studies have assessed the effectiveness of different surgical procedures. For cutaneous melanoma, wide tumour excision with a 2-centimetre margin is recommended (11–13). However, most mucosal melanomas are not suitable for wide tumour excision. There is a lack of consensus regarding the benefits of radical resection. Some studies have suggested that there is no benefit to more radical surgical approaches, as they do not improve patient survival. In one study of 22 patients, half of the patients underwent radical surgery, and the other half underwent conservative surgery. The results showed that radical resection is unlikely to improve the prognosis of patients (5). In another study, radical surgery did not improve or worsen the survival of patients (7). Few studies (6) with small sample sizes reached the same conclusions. These findings are consistent with our research showing that radical resection is not an independent prognostic factor for OS.

However, the above studies did not mention whether radical resection can improve RFS. One study demonstrated that the 5-year survival rate was unrelated to the type of therapy but that radical surgery appeared to control local disease (14). In our study, the surgical approach was associated with RFS according to the multivariate analysis, which corrected for potential confounding factors. RFS was longer in the radical resection group than in the non-radical resection group (mRFS 9.5 vs. 5.3 m) (Figure 1A). To identify better subgroups for RFS between the two groups, we carried out univariate Cox regression analyses, and we did not observe that a particular subgroup was associated with RFS (Figure 6).

Studies have demonstrated that the depth of tumour invasion is an important prognostic factor of mucosal melanomas (15). The cancer staging criteria for cutaneous melanoma include tumour thickness (mm) for assigning T stage (8, 16). Owing to mucosal anatomy, a specific depth is not suitable for staging mucosal melanoma. Similar to the staging criteria for mucosal melanoma of the head and neck, T stage is defined according to whether the tumour is limited to the mucosa or has invaded the immediately underlying soft tissue; it is not defined according to the thickness or greatest dimension of the tumour (8). Hence, in our study, patients were divided into five subgroups: mucosa, muscular layer, serous membrane, adjacent structures, and unknown.

Our study found that lymph node status was associated with RFS but not OS. Only a minority of patients had positive metastatic lymph nodes. This finding may be the main cause of the difference from previous studies. CLND was irrelevant to RFS and OS. Lymphatic mapping with sentinel lymph node biopsy (SLND) is the standard treatment for cutaneous melanoma (17–19) when feasible. The phase III MSLT-II trial included 1,934 evaluable patients to assess the effectiveness of CLND for patients with sentinel lymph node (SNL) metastases (20). The results showed that CLND did not increase melanoma-specific survival among patients with melanoma and sentinel-node metastases, which is in agreement with the results of the DeCOG-SLT trial (19). Lymphatic drainage is quite complex in gynaecologic organs, and SLNs may be in inguinal basins, pelvic basins, or both (21–23). The conditions under which SLND should be performed for gynaecological melanoma remain debated (21, 24).

In our review, a large proportion of the patients were vaginal melanoma, the results is consistent with the previous literatures (25). Cervical melanoma is a very rare histopathologic subtype of primary cervical cancer. In a few case reports, cervical melanoma was managed similarly to cervical carcinoma (26–29). For curative-intent treatment, radical resection is the main surgical approach. In our study, only two patients were treated with non-radical resection.

The common perioperative complications of gynaecological operations are infection, bladder and ureteral injuries, gastrointestinal dysfunctions, and fistulas (30–32). Other complications, such as haemorrhage, deep vein thrombosis, fluid or electrolyte imbalance, and pneumonia, are common in all types of surgeries. Thirty-eight patients died or did not undergo surgery in our centre. Therefore, the information about complications were incompeted. Five patients developed post-operative complications. Among them, two patients who underwent non-radical resection experienced delayed operative incision healing. In the radical resection group, one patient experienced delayed operative incision healing, one patient experienced bowel obstruction and urinary retention, and one patient experienced urinary retention. In the past several decades, the development of surgical techniques and methods of perioperative care, such as reconstructive surgery of the perineum (33, 34), the application of advanced surgical instruments and the implementation of enhanced recovery after surgery (ERAS) pathways (35–37), has decreased perioperative complications and increased patient quality of life.

In our study, we found that adjuvant therapy significantly prolonged RFS and OS for patients with mucosal melanoma. The most common treatments, in descending order, were chemotherapy (39%), IFN (21%) and immunotherapy (10%) (Figure 4A). In a phase II randomised trial, temozolomide-based chemotherapy and HDI prolonged RFS and OS among patients with completely resected mucosal melanoma (38). Some phase III trials, including the Eastern Cooperative Oncology Group (ECOG) E1609 trial (39), Checkmate 238 trial (40), and Keynote-054 trial (41), found that immunotherapy as adjuvant therapy for patients with resected high-risk cutaneous melanoma can significantly prolong RFS. Some of the trials (including ECOG E1609 and Checkmate 238) enrolled patients with mucosal melanoma but did not report details about treatment effectiveness for the mucosal melanoma subgroup.

In this study, after recurrence, the majority of patients (39%) had received combined treatment (Figure 4B), including chemotherapy combined with antiangiogenic therapy and immunotherapy combined with antiangiogenic therapy. A considerable proportion of the patients (17%) underwent a second surgery, and the mRFS after reoperation was 4.4 months. Because most of the patients who underwent palliative therapy failed to complete the treatment in our centre, mPFS data were not available for these patients. Immunotherapy significantly improved PFS for patients with advanced cutaneous melanoma (42–44). However, mucosal melanoma has completely different biological behaviours from cutaneous melanoma. Compared with cutaneous melanoma, mucosal melanoma can infiltrate earlier in the disease process and travel faster through the lymphatic and vascular systems. Some exploratory trials showed that chemotherapy combined with antiangiogenic therapy and immunotherapy combined with antiangiogenic therapy improved survival for patients with advanced mucosal melanoma (45, 46).

The strengths of our study are the considerable follow-up time and complete data, and all the data were from the real world, providing real-world evidence. We selected RFS and OS as the endpoints in this study, which can reflect the short-term and long-term outcomes of surgical procedures. Certainly, our study has several limitations. First, it was a retrospective study with unavoidable inherent bias. We strictly followed the principles and methods for observational studies and consecutively identified as many patients as possible to try our best to reduce information bias and selection bias. In addition, we carried out multivariate Cox proportional hazards models to reduce confounding bias. Second, four patients were lost to follow-up; therefore, the effectiveness of the operation and the survival outcomes of these patients could not be evaluated. Third, thirty-eight patients died or did not recieve surgery in our centre; therefore, the information about complications were incomplete. Finally, although our study included a large number of patients compared with the numbers in some previous studies and focused on the effectiveness of different surgical approaches, the sample size of our study was small.

In conclusion, we did not identify a survival benefit of radical surgery in patients with vaginal melanoma and cervical melanoma. The addition of adjuvant therapy prolonged both RFS and OS in patients.

## Data Availability Statement

The raw data supporting the conclusions of this article will be made available by the authors, without undue reservation.

## Ethics Statement

The studies involving human participants were reviewed and approved by Institutional Review Boards of Peking University Cancer Hospital. The patients/participants provided their written informed consent to participate in this study.

## Author Contributions

CC: research concept and design. HT and XW: data collection, data analysis and interpretation, and manuscript writing. All authors read and approved the final manuscript.

## Funding

This work was supported by Grant No. 81972562 from the National Natural Science Foundation of China.

## Conflict of Interest

The authors declare that the research was conducted in the absence of any commercial or financial relationships that could be construed as a potential conflict of interest.

## Publisher's Note

All claims expressed in this article are solely those of the authors and do not necessarily represent those of their affiliated organizations, or those of the publisher, the editors and the reviewers. Any product that may be evaluated in this article, or claim that may be made by its manufacturer, is not guaranteed or endorsed by the publisher.
